# Ratio of Intratumoral Macrophage Phenotypes Is a Prognostic Factor in Epithelioid Malignant Pleural Mesothelioma

**DOI:** 10.1371/journal.pone.0106742

**Published:** 2014-09-05

**Authors:** Robin Cornelissen, Lysanne A. Lievense, Alexander P. Maat, Rudi W. Hendriks, Henk C. Hoogsteden, Ad J. Bogers, Joost P. Hegmans, Joachim G. Aerts

**Affiliations:** 1 Department of Pulmonary Medicine, Erasmus MC Cancer Institute, Rotterdam, The Netherlands; 2 Department of Cardio-Thoracic Surgery, Erasmus MC Cancer Institute, Rotterdam, The Netherlands; University of Pittsburgh, United States of America

## Abstract

**Hypothesis:**

The tumor micro-environment and especially the different macrophage phenotypes appear to be of great influence on the behavior of multiple tumor types. M1 skewed macrophages possess anti-tumoral capacities, while the M2 polarized macrophages have pro-tumoral capacities. We analyzed if the macrophage count and the M2 to total macrophage ratio is a discriminative marker for outcome after surgery in malignant pleural mesothelioma (MPM) and studied the prognostic value of these immunological cells.

**Methods:**

8 MPM patients who received induction chemotherapy and surgical treatment were matched on age, sex, tumor histology, TNM stage and EORTC score with 8 patients who received chemotherapy only. CD8 positive T-cells and the total macrophage count, using the CD68 pan-macrophage marker, and CD163 positive M2 macrophage count were determined in tumor specimens prior to treatment.

**Results:**

The number of CD68 and CD163 cells was comparable between the surgery and the non-surgery group, and was not related to overall survival (OS) in both the surgery and non-surgery group. However, the CD163/CD68 ratio did correlate with OS in both in the total patient group (Pearson r −0.72, p<0.05). No correlation between the number of CD8 cells and prognosis was found.

**Conclusions:**

The total number of macrophages in tumor tissue did not correlate with OS in both groups, however, the CD163/CD68 ratio correlates with OS in the total patient group. Our data revealed that the CD163/CD68 ratio is a potential prognostic marker in epithelioid mesothelioma patients independent of treatment but cannot be used as a predictive marker for outcome after surgery.

## Introduction

Malignant pleural mesothelioma is invariably a lethal tumor with a median survival of 9–12 months after the first signs of illness. It is one of the diseases caused by exposure to asbestos fibers. The incidence varies from two to 30 cases per 1 000 000 population worldwide. Most patients are older than 60 years, a reflection of the latency period of 30–50 years after asbestos fiber inhalation.

Chemotherapy is offered to patients as standard of care treatment, as it currently is the only treatment that improved survival in randomized controlled trials in mesothelioma patients [1], [2]. The survival benefit of chemotherapeutic treatment is in general modest with 2–3 months but long-term survivors do exist.

For decades, clinicians have tried to improve survival by removal of the pleural-based lesions. In order to try to completely remove the disease, a pneumonectomy with the complete removal of the visceral and parietal pleura is considered necessary, a so-called extra-pleural pneumonectomy (EPP). EPP is mostly performed in a multi-modality setting with induction chemotherapy and adjuvant radiotherapy. Selection of patients appeared crucial in the case-series that were published [3]. A less invasive procedure, that does not include the removal of the affected lung but of the visceral and parietal pleura, if necessary pericardium and diaphragm, an extended pleurectomy/decortication (PD), is also performed in patients.

Whether surgery does lead to increased survival remains a matter of continuous debate, but it is evident that long-term survival after surgery occurs [4], [5]. On the other hand, there are also patients in whom survival after surgery is extremely short. This points out the need for a biomarker to provide insight in which patients may benefit from surgery and which patients do not.

Gordon *et al.* described a four-gene expression ratio test that can predict good prognosis after surgery [6], however this test still has to be validated in a clinical setting. Suzuki *et al.* found in a patient group with predominantly surgical therapy that chronic inflammation in stroma is an independent predictor of survival [7], while other groups found a subset of immunological cell types to predict for better outcome in patients receiving surgical treatment with a special focus on CD8 tumor infiltrating lymphocytes [8], [9]. The question remains whether these factors are prognostic or predictive for the effect of surgery.

The role of immune cells, like CD8 cells, within the tumor microenvironment has become a major area of interest in the last decade. It is now established in certain tumor types, that these infiltrating immune cells are capable of influencing tumor progression. One of the other involved immunological cell types are macrophages, which are known to have a dual role in cancer depending on their phenotype. Tumor associated macrophages (TAMs) can be divided in classically activated (M1) macrophages and alternatively activated macrophages (M2). M1 macrophages, following exposure to interferon-γ (IFN-γ), can secrete chemokines and promote T cell proliferation, thus activate type 1 T cell responses and have antitumor activity and tissue-destructive activity. However, M2 TAMs promote the development and metastatic capacity of tumors due to the production of multiple cytokines such as interleukin (IL)-1, IL-6 and IL-10, vascular endothelial growth factor (VEGF) and transforming growth factor beta (TGF-β) [10]. In mesothelioma, Burt *et al* showed that higher densities of tumor-infiltrating macrophages are associated with poor survival in patients after surgery, however, this was only in patients with non-epithelioid MPM [11].

A large proportion of M1 macrophages in the total macrophage count that can aid in tumoricidal activities could provide a better tumor control, since the overall balance in the tumor microenvironment shifts to an anti-tumor response. If the TAMs largely consist of M2 macrophages, this balance can shift to an overall pro-tumor micro-environment. The importance of the percentage of M2 macrophages of the total macrophage count (i.e. the CD163/CD68 ratio) and M1/M2 ratio has been found in other tumor types recently, such as melanoma, non-small cell lung carcinoma and angioimmunoblastic T-cell lymphoma [12]–[17]. In most of these studies, the ratio of M1/M2 macrophages predicts survival and metastatic ability of these cancers. Overall, a larger M2 component of the total macrophage count is inversely correlated with survival.

With CD8 T-cells and TAMS being the key immune cells in the tumor microenvironment [18], [19], we analyzed if T cells and macrophage subtypes could be useful as a predictive marker to select mesothelioma patients for surgical treatment. Furthermore, the prognostic value of the different macrophage subtypes and CD8 positive tumor infiltrating lymphocytes (TILs) were tested.

## Materials and Methods

### Patients and specimens

The Erasmus Medical Center ethical commission gave approval for this study. Diagnostic paraffin-embedded tumor specimens were used from 8 MPM patients who underwent an extended PD during the course of a phase l clinical trial following induction chemotherapy in our institute between 2008 and 2010 (a local study which is identified as Erasmus MC Cancer Institute MEC number 2008-405). The clinical trial randomized patients to P/D or best supportive care. Consent was obtained to use patient material for future research. Unfortunately, from the patients randomized to the best supportive care arm, adequate histology was not available in all cases. Therefore, we selected 8 MPM out of the total 89 patients that only were treated with chemotherapy during the course of the trial. The selection was matched to the surgical cases upon survival, EORTC prognostic score [20] and histology. Patient information was anonymized end de-identified prior to analysis. Histopathological diagnoses were established by pathologists from our institute and confirmed by the National Mesothelioma Pathology Board. Clinicopathological information was collected from patient charts. The TNM stage was based on the International Union Against Cancer (UICC) and the American Joint Committee on Cancer (AJCC) classification. Overall survival (OS) analysis of patients who underwent either chemotherapy or chemotherapy and PD was conducted. OS was defined as the time from the completion of chemotherapy to death. Three patients are still alive at the time of submitting this manuscript, since these are the 3 patients with the longest survival, last contact date was used instead of date of death.

### Immunohistochemistry

The following primary antibodies were used: anti-human CD8 (clone C8/144B, Dako, Glostrup, Denmark), anti-human CD68 (clone KP-1, Dako), and anti-human CD163 (clone 10D6,Leica Biosystems Novocastra, Newcastle, UK). Paraffin-embedded tumor specimens were cut into sequential 5 µm thick sections and deparaffinized and stained using a fully automated Ventana BenchMark ULTRA Stainer (Ventana, Tucson Arizona, USA) according to manufacturers' instructions at the pathology department. Binding of peroxidase-coupled antibodies was detected using 3,3′ - diaminobenzidine (DAB) as a substrate and the slides were counterstained with haematoxylin. The specificity of antibodies was checked using isotype-matched controls.

### Evaluation of CD8, CD68 and CD163 stainings

The number of CD8-positive T-cells, CD68-positive total macrophages and CD163-positive M2-type macrophages were independently assessed by two investigators (R.C. and L.L.) who were not informed of the patients' clinicopathological data. To examine TILs and TAMs, the number of cells per microscopic field of 0,025 cm^2^ with immunoreactivity to CD8, CD68 and CD163 were counted in three independent tumor areas with the most abundant immunoreactive cells. For each antibody, the same area was used. Only cells with a visible nucleus were counted. We defined the average value of the three times the number of TILs and TAMs were counted for each case.

### In vitro measurement of CD80, HLA-DR, IL-10, IL-12, VEGF, PD-L1, CD163, iNOS (NOS2) and Arginase-1 in macrophages by quantitative real time PCR

We investigated the influence of mesothelioma-derived factors on the phenotype and function of macrophages. Monocytes obtained from peripheral blood of an healthy control were cultured in the presence of 20 ng/ml recombinant M-CSF (R&D systems, Abingdon U.K.) in RPMI medium (Life Technologies, Bleiswijk, the Netherlands) containing 5% normal healthy AB serum (NHS) during 6 days at 37°C/5% CO_2_. After six days of differentiation, macrophages were cultured in the presence of 30% mesothelioma cell line conditioned media (CM) during two days (n = 6). CM were obtained from mesothelioma cell lines at 80% confluency, centrifuged for 10 min at 400×g to remove cells and debris. These long-term tumor cell lines were established from the cellular fraction of 6 mesothelioma patient's pleural effusions as described earlier [21]. As a control we used standardized M1 (medium supplemented with 100 ng/ml LPS [Sigma-Aldrich, Zwijndrecht, the Netherlands] and 20 ng/ml IFN-gamma [R&D systems) and M2 cultures (medium supplemented with 40 ng/ml IL-10 [R&D systems]). Cells were harvested and mRNA was isolated by RNeasy micro kit according to manufacturer's instruction (Qiagen, Hilden, Germany). cDNA was prepared from 1 ug RNA sample using First Strand cDNA synthesis kit (Thermo Fisher, Pittsburgh, PA, USA). cDNA (5 µL) was amplified by RT-PCR reactions with 1× Maxima SYBR green/ROX qPCR mastermix (Thermo Fisher) in 96-well plates on an 7300 real time PCR system (Applied Biosystems), using the program: 10 min at 95°C, and then 40 cycles of 20 s at 95°C, 1 min at 58°C and 30 sec at 72°C. The primer sets used for different sets of genes are listed in Table 1. Specificity of the produced amplification product was confirmed by examination of dissociation curves. Expression levels were normalized to the internal control β-actin.

**Table 1:** Primer sequences of genes associated with macrophage phenotype used in RT-PCR.

Gene	Forward primer	Reverse primer
*β*-actin	CTGTGGCATCCACGAAACTA	AGTACTTGCGCTCAGGAGGA
CD80	AAACTCGCATCTACTGGCAAA	GGTTCTTGTACTCGGGCCATA
HLA-DR	AGTCCCTGTGCTAGGATTTTTCA	ACATAAACTCGCCTGATTGGTC
IL-10	TCAAACTCACTCATGGCTTTGT	GCTGTCATCGATTTCTTCCC
IL-12	GCGGAGCTGCTACACTCTC	CCATGACCTCAATGGGCAGAC
VEGF	CACACAGGATGGCTTGAAGA	AGGGCAGAATCATCACGAAG
PD-L1	TATGGTGGTGCCGACTACAA	TGCTTGTCCAGATGACTTCG
CD163	GCGGGAGAGTGGAAGTGAAAG	GTTACAAATCACAGAGACCGCT
iNOS	ATTCTGCTGCTTGCTGAGGT	TTCAAGACCAAATTCCACCAG
Arg1	GTTTCTCAAGCAGACCAGCC	GCTCAAGTGCAGCAAAGAGA

### Statistical analysis

The numbers of CD8 TILs and CD163 and/or CD68 TAMs were expressed as mean ± SD. Statistical differences between the means were analyzed by the Mann–Whitney U test. Correlations were made calculating the Pearson r correlation. Statistical calculations were performed using IBM SPSS Statistics version 21.0.0.1. Statistical significance was established at the p<0.05 level, and all analyses were two-sided. Overall survival (OS) was calculated from the start date of treatment until patient death.

## Results

### Patient characteristics

The median age of all participating patients was 62 years (range 36-75 years). There were 12 men and 4 women. All histologies were of the epithelioid subtype. The patient characteristics of the surgery and the non-surgery group are listed in Table 2. Chemotherapeutic treatment was given in both groups and consisted of 4 cycles of pemetrexed combined with either cisplatin or carboplatin. In case of surgery, P/D was performed 8 to 10 weeks after induction chemotherapy in all cases.

**Table 2:** Patient characteristics.

	Surgery	Non-surgery
Patients (n)	8	8
Mean age (SD)	60 (11,9)	55 (7)
Male (n)	6	6
EORTC (SD)	1,025 (0,6)	0,88 (0,5)
EORTC high (n)	2	1
EORTC low (n)	6	7
PR after chemotherapy (n)	1	2
TNM		
T1-2 (n)	6	5
T3-4 (n)	2	3
N0 (n)	5	5
N1-2 (n)	3	3
M0 (n)	8	7

### CD8 tumor infiltrating lymphocytes in MPM

**Figure 1:**
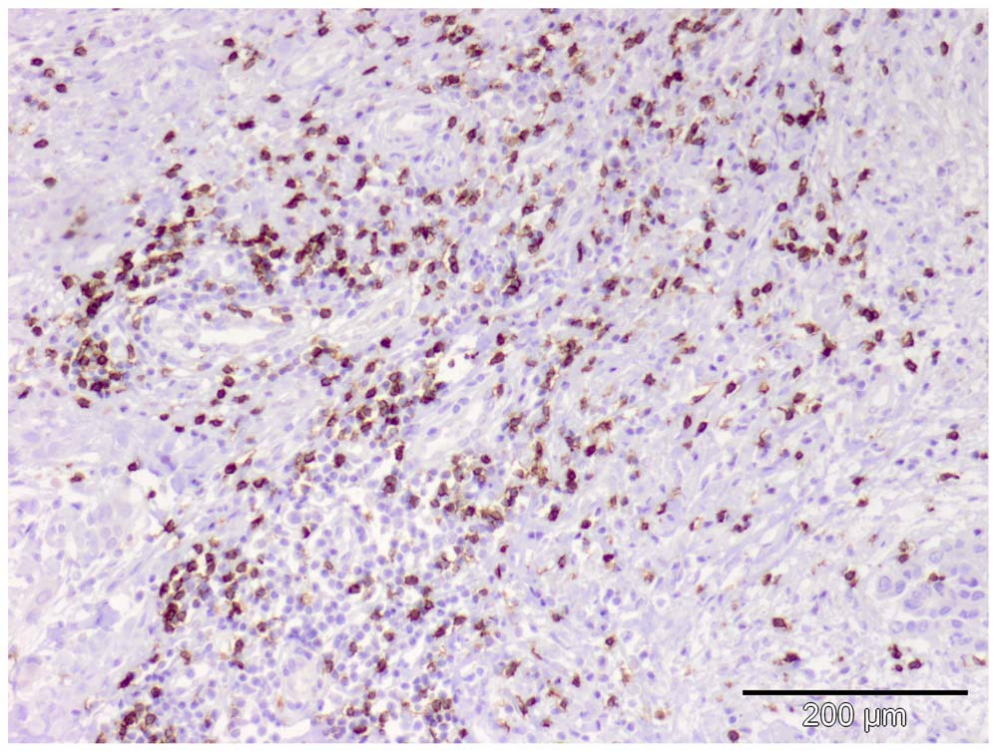
Representative image of CD8 staining in the tumor biopsy of one MPM patient.

A representative image of immunohistochemical staining of CD8 TILs are shown in Figure 1. The mean CD8 numbers were comparable between the surgery and the non-surgery group (p = 0.51) and no correlation was found between CD8 cell count and OS in the surgery group (p = 0.88) and non-surgery group (p = 0.96) nor for the whole group (p = 0.73).

### CD68 and CD163 TAMs in MPM

**Figure 2:**
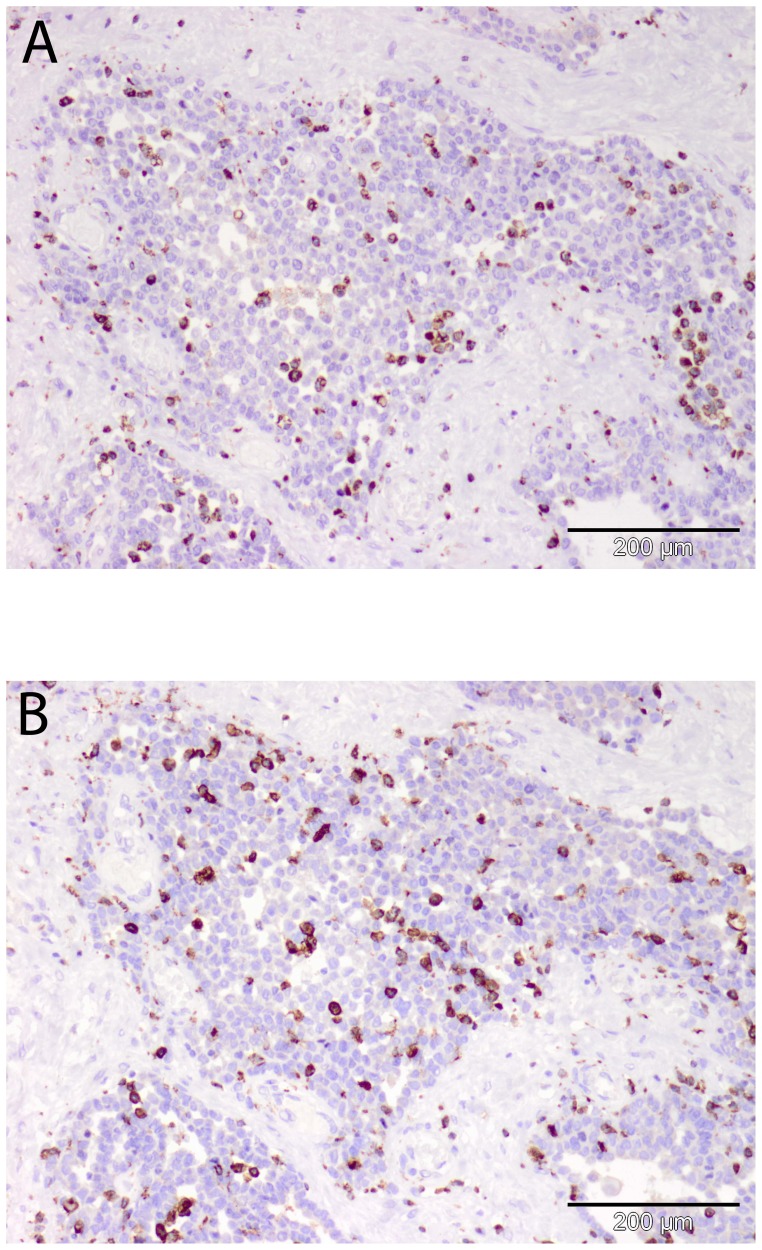
Representative images of CD68 (a) and CD163 (b) staining in the tumor biopsy of one MPM patient.

Representative images of immunohistochemical staining of TAMs are shown in Figure 2a and 2b. The total count of CD68 was comparable between surgery and the non-surgery group (mean 211.3, SD 80.2 vs. mean 213.9, SD 100.4, p = 1.0). Also, the total count of CD163 was comparable between surgery and the non-surgery group (mean 168.3, SD 80.2 vs. mean 164.1, SD 82.5, p = 0.8).

**Figure 3:**
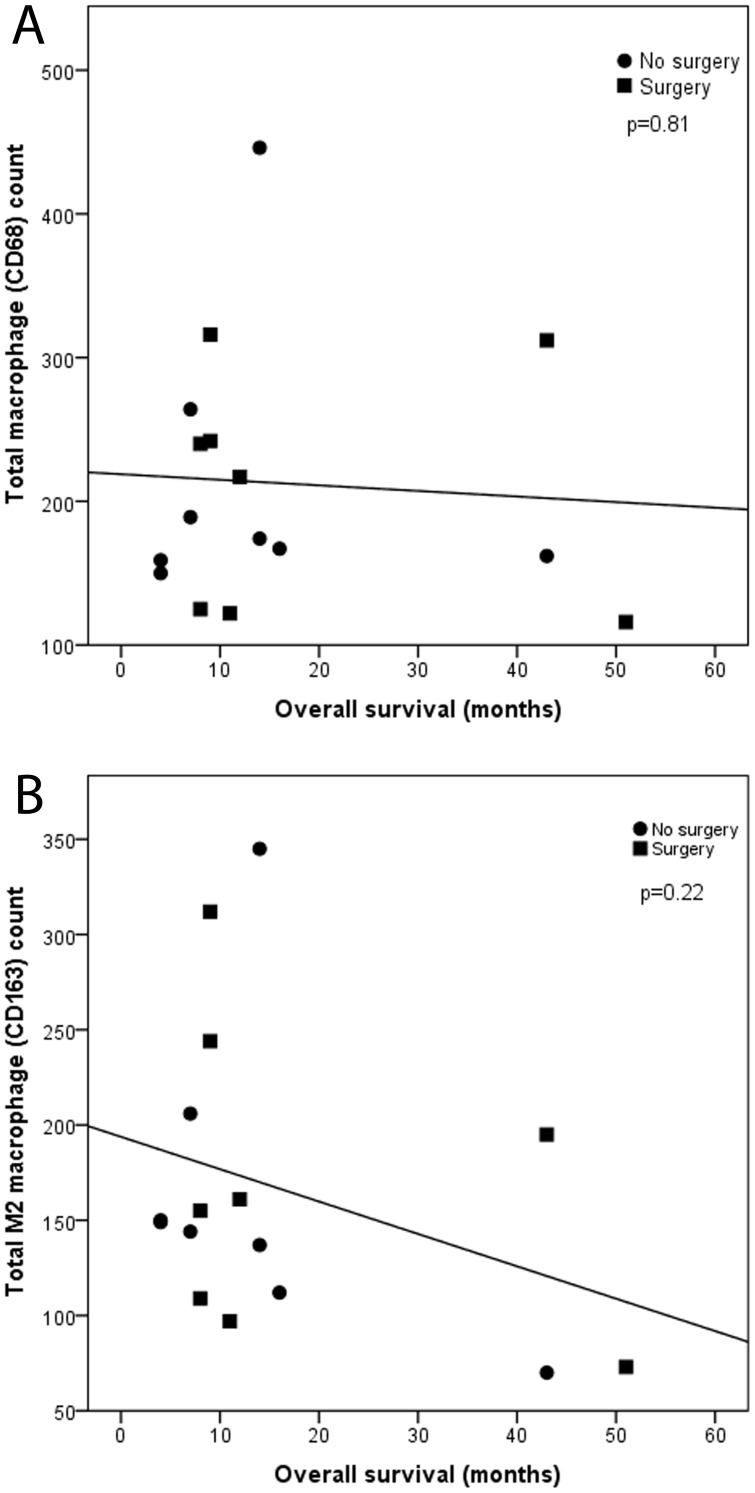
Correlation between CD68 (a) count or CD 163 (b) count and OS in both surgery and non-surgery groups. The CD68 count does not correlate with OS (Pearson r -0.07, p = 0.81), the CD163 count shows an inverse trend with OS (Pearson r -0.33, p = 0.22).

The CD68 count did not correlate with OS (Figure 3a, Pearson r -0.07, p = 0.81), the CD163 count showed an inverse trend with OS (Figure 3b, Pearson r -0.33, p = 0.22).

### CD163/CD68 ratio correlating with overall survival

**Figure 4:**
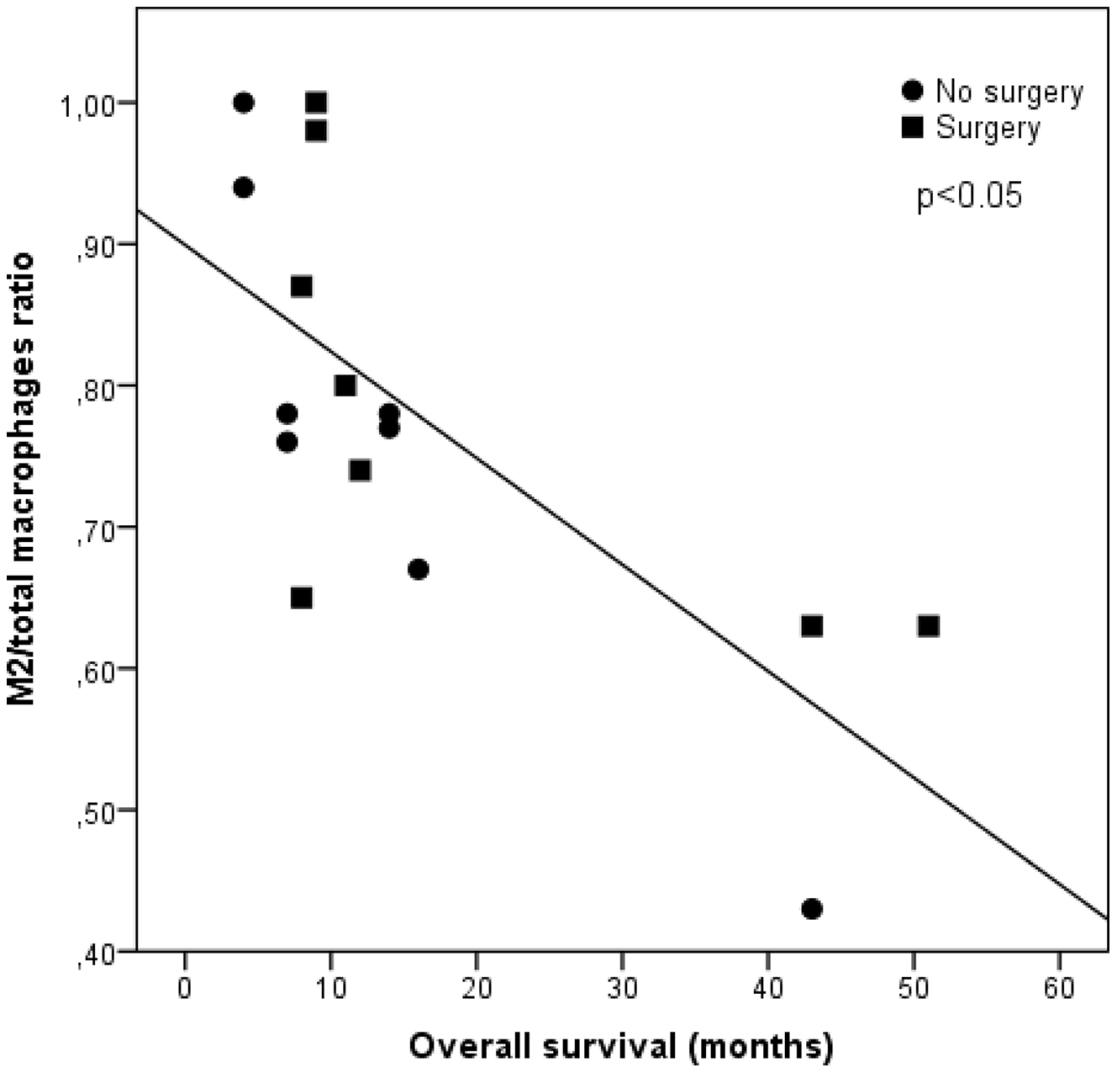
Correlation between CD163/CD68 ratio in tumor in both surgery and non-surgery patients and OS. This ratio is significantly negatively correlated with OS in the total patient group (Pearson r -0.72, p<0.05).

We calculated the CD163/CD68 ratio, i.e. the number of M2 macrophages within the total macrophage count. This ratio was significantly negatively correlated with OS in the total patient group (Figure 4, Pearson r -0.72, p<0.05). A correlation analysis for the individual groups in regards to the CD163/CD68 and OS showed a significant correlation in the non-surgery group (Pearson r -0.91 [p = 0.001]) and a trend for the surgery group (Pearson r -0.65 [p = 0.08]).

### RT-PCR measurements for macrophage phenotype conditioned in mesothelioma environments

**Figure 5:**
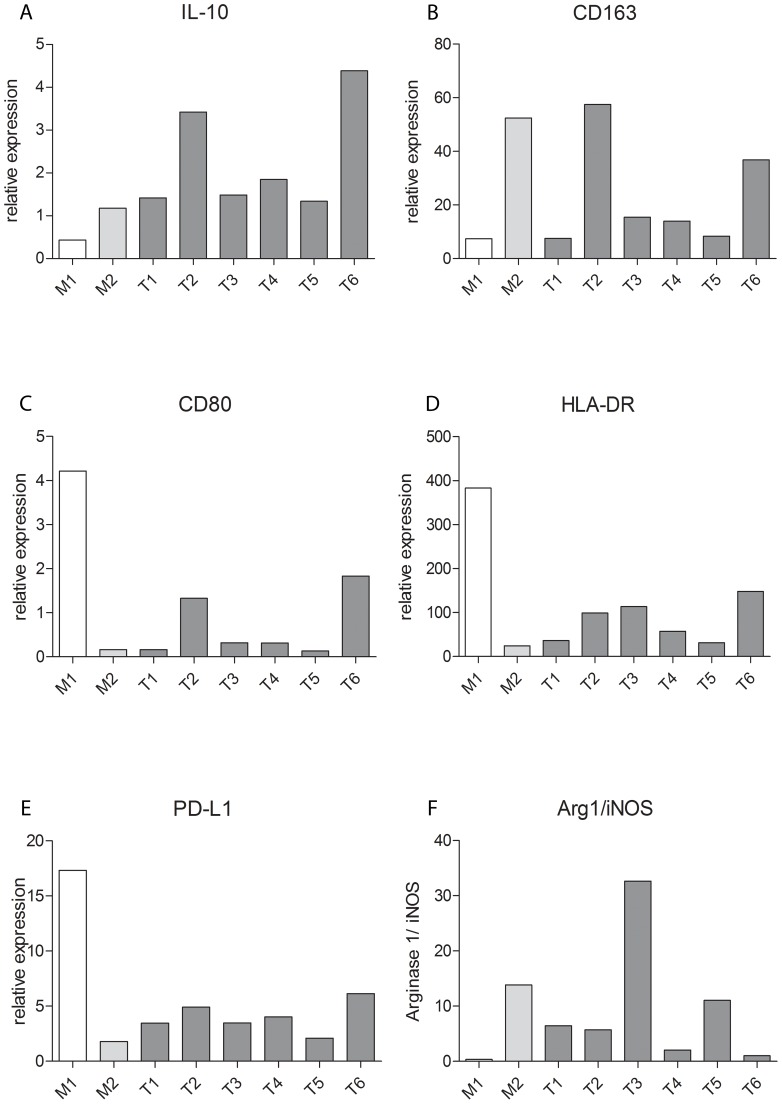
Tumor derived factors influence macrophages towards a M2 phenotype to varying degrees. Relative mRNA expression levels of IL-10 (a), CD163 (b), CD80 (c), HLA-DR (d), PD-L1 (e), and Arginase-1/iNOS (NOS2) ratio (f) in macrophages cultured in six mesothelioma cell line conditioned media (T1 - T6) compared to standard M1 and M2 conditions.

To investigate the influence of tumor-derived factors on macrophage phenotype, we cultured monocyte-derived macrophages in the presence of supernatant derived from six mesothelioma cell lines. Tumor cell supernatants (CM) induced macrophages towards a M2 prone phenotype with relatively high expression levels of the M2 cytokine IL-10 and low mRNA levels of the M1 markers IL-12, CD80 and HLA-DR. The standard M2 marker CD163 and the arginase1/iNOS ratio showed differential expressions dependent on the different CM. Furthermore, expression levels of the activation marker PD-L1 on macrophages cultured in CM were comparable to the M2 condition, in general these levels were lower than the M1 condition. Furthermore, results showed that CM have different abilities to influence macrophage phenotypes (Figure 5). Gene expression of IL-12 was only found when macrophages were cultured under M1 conditions and VEGF expression was low/absent in all conditions (data not shown). In conclusion, mesothelioma-derived factors influence macrophages towards a M2 phenotype to varying degrees.

## Discussion

Macrophages in tumors are usually referred to as tumor-associated macrophages and their presence can be substantial (up to 60% of the tumor mass) [22]. A hallmark of macrophages is their plasticity, an ability to either aid or fight tumors depending on the tumor environment, which has given them the reputation of a double-edged sword in tumor biology [23]. At the extremes of this spectrum are the M1 and M2 macrophages. In an early phase of tumor development, the TAMs mainly consist of an M1-like phenotype and later in the tumorigenic process, when the tumor changes its local environment, there is a skewing toward the M2 phenotype [24]–[26]. Analysis of CD163/CD68 ratio in biopsy material before treatment showed a correlation with OS (combined groups: Pearson r -0.72 [p<0.05]; non-surgery group: Pearson r -0.91 [p = 0.001]; surgery group: Pearson r -0.65 [p = 0.08]). The total number of macrophages did not correlate with OS, indicating that the absolute number of macrophages does not influence tumor progression. The percentage of M2 macrophages of the total macrophage count was comparable between the surgery and non-surgery group and therefore, the CD163/CD68 ratio does not discriminate in favor of surgery in mesothelioma patients.

Although the terms M1 and M2 macrophages are an oversimplification of reality, it can be used to explain the opposing effects of different macrophage subsets. Our findings indeed correspond with the negative prognostic capacities of the M2 macrophages; a large proportion of these CD163 positive macrophages in the total macrophage count correlates with a decreased survival. This emphasis that the balance between M1 and M2 macrophages seems to play a crucial role in the prognosis of MPM patient.

As mentioned before, the importance of the CD163/CD68 and M1/M2 ratio is found in several other tumor types [12]–[17]. In our study, a similar outcome is found regarding M1/M2 ratio based on CD163/68 ratio and the prediction of survival in patients with mesothelioma. This gives a clinical correlation to the hypothesis of the anti-tumor effect of M1 TAMs and the pro-tumor effect of the M2 TAMs. To our knowledge, this is the first publication showing the importance of the CD163/CD68 ratio in mesothelioma. Furthermore, this ratio proved to be significantly correlated with survival in epithelioid mesothelioma. Previously, it was only shown that the absolute number of macrophages was prognostic in non-epithelioid mesothelioma after EPP [11].

In previous studies looking at the number of CD8 TIL's a high number of CD8 TIL was associated with a better outcome in mesothelioma patients after surgery [8], [9]. We could not reproduce these findings in our study. This could be due to the smaller numbers of surgical patients that were available for our study. Furthermore, the correlation between TIL count and survival was only found in patients that received chemotherapy and EPP, while in our study, P/D was performed.

The six mesothelioma cell lines showed evident heterogeneous effects on the macrophages in terms of macrophage polarization. Tumor-derived factors from cell lines induced M1 and M2 macrophage phenotypes in varying degrees, in concordance with the broad phenotype spectrum found in tumors. However, overall the tumor cell supernatants induced a more M2 prone phenotype with relatively high expression levels of IL-10 and low expression levels of M1 markers: IL-12, CD80 and HLA-DR. The standard M2 marker CD163 and the arginase1/iNOS ratio showed very differential results between the tumor cell lines. Furthermore, PD-L1 expression levels appeared to be relatively low. However, PD-L1 is known to be upregulated in a response to high IFN-γ levels as a negative feedback mechanism and therefore although PD-L1 is a co-inhibitory receptor, its presence can be indicative of an active T-cell response [27]–[29]. This was confirmed by the high PD-L1 level in the M1 condition. The *in vitro* experiments using tumor derived factors to influence macrophage phenotype complement the *in vivo* immunohistochemical findings by demonstrating that tumor-derived factors can directly modulate macrophage phenotype multiformity.

In addition to the impact of this finding on prognostic value of the OS of patients, macrophages may also reveal as a potential target for therapeutic intervention. Targeting the total macrophage population would not be the most optimal approach, since M1 macrophages would be decreased as well as the M2 macrophages. In an earlier trial we showed that this kind of intervention does not lead to increased survival in a murine model of mesothelioma [36]. There are several proposed strategies to counteract the M2 macrophages, including inhibiting M2 macrophage recruitment [37], M2 macrophage depletion [38] and blocking M2 tumor-promoting activity of TAMs [39]. However, since M2 macrophages remain the plasticity for polarization [40], re-polarization from M2 to M1-type could be the ideal method to tip the balance between M1 and M2 to a tumor-hostile situation. Recently, it has become clear that there is probably not one single compound that can achieve this goal [22]. A proposed strategy therefore is a combination of infusion of antibodies against CD40 in order to stimulate the secondary lymph node resident macrophages to migrate into the tumor tissue with IFN-γ to effectively reprogram tumor-induced M2-like macrophages into activated IL-12 producing M1 cells [41]. In addition, targeting the nuclear factor κB (NF-κB) signaling pathway, a crucial pathway in the activation of M2 TAMs, was shown to switch M2 TAMs to a M1 phenotype [42]. Furthermore, the combined use of Toll-like receptor 9 ligand CpG-ODN and anti-IL-10 blocking antibodies has been shown to induce the switch from M2 to M1 phenotype [43]. Also, several other therapeutic strategies are under investigation [44]–[47]. In mesothelioma, Fridlender et al. tested monocyte chemoattractant protein-1 (MCP-1/CCL2) blockade in a mouse model for mesothelioma and demonstrated an altered macrophage phenotype and improved survival. Currently there are no clinical compounds tested in mesothelioma patients which specifically aim at macrophage repolarization [48].

Our study has several limitations. First, the number of patients included is rather small. This is due to the fact that mesothelioma surgery in Europe is advised to be only performed in the setting of a clinical trial by the guidelines of the European Respiratory Society and the European Society of Thoracic Surgeons for the management of malignant pleural mesothelioma [30]. The results of the present trial are based on a trial randomizing patients between P/D or observation. This trial was stopped based on slow accrual. Furthermore, only patients with the epithelioid subtype of mesothelioma were selected for surgery. The trend seen in the surgery group between the CD163/CD68 ratio and OS should be confirmed in a larger patient group and we hope that our findings will encourage other researchers who have access to patients undergoing surgery to confirm the data presented in this manuscript. Second, a definitive M1 macrophage marker would enhance the findings of our manuscript for this would give a true insight in the M1/M2 macrophage ratio. NOS2 expression has proven be a useful marker for M1 macrophages in several tumor types [31]–[33]. However, for mesothelioma, Soini et al. and others [34], [35] have demonstrated that NOS2 is highly expressed in healthy pleura as well as in cancerous mesothelioma tissues and mesothelioma cell lines. These findings complicate the use of NOS2 in pleural diseases as mesothelioma. Whether the unique capacity of mesothelial/mesothelioma tumor cells of synthesizing NOS2 is important to control a variety of infections in the pleural space in particular is unknown.

In conclusion, CD163/CD68 ratio was found to be a prognostic marker in a limited number of epithelioid mesothelioma patients, but not a predictive marker for outcome after surgery. This study emphasizes the importance of the balance between M1 and M2 macrophages in tumor behavior. In spite of not being a predictive factor for surgery in mesothelioma, we consider that the prognostic value may be of great importance in patients with mesothelioma. Repolarization of macrophages may be a new therapeutic target in mesothelioma complementing immunotherapeutic strategies.
